# Physicochemical, Pharmacokinetic, and Toxicity Evaluation of Methoxy Poly(ethylene glycol)-*b*-Poly(d,l-Lactide) Polymeric Micelles Encapsulating Alpinumisoflavone Extracted from Unripe *Cudrania tricuspidata* Fruit

**DOI:** 10.3390/pharmaceutics11080366

**Published:** 2019-08-01

**Authors:** Min Jeong Jo, Yang Hee Jo, Yu Jin Lee, Chun-Woong Park, Jin-Seok Kim, Jin Tae Hong, Youn Bok Chung, Mi Kyeong Lee, Dae Hwan Shin

**Affiliations:** 1 College of Pharmacy, Chungbuk National University, Cheongju 28160, Korea; 2 Drug Information Research Institute (DIRI), College of Pharmacy, Sookmyung Women’s University, Seoul 04310, Korea

**Keywords:** alpinumisoflavone, mPEG-*b*-PLA micelle, solubilization, pharmacokinetics, toxicity

## Abstract

Alpinumisoflavone, a major compound in unripe *Cudrania tricuspidata* fruit is reported to exhibit numerous beneficial pharmacological activities, such as osteoprotective, antibacterial, estrogenic, anti-metastatic, atheroprotective, antioxidant, and anticancer effects. Despite its medicinal value, alpinumisoflavone is poorly soluble in water, which makes it difficult to formulate and administer intravenously (i.v.). To overcome these limitations, we used methoxy poly(ethylene glycol)-*b*-poly(d,l-lactide) (mPEG-*b*-PLA) polymeric micelles to solubilize alpinumisoflavone and increase its bioavailability, and evaluated their toxicity in vivo. Alpinumisoflavone-loaded polymeric micelles were prepared using thin-film hydration method, and their physicochemical properties were characterized for drug release, particle size, drug-loading (DL, %), and encapsulation efficiency (EE, %). The in vitro drug release profile was determined and the release rate of alpinumisoflavone from mPEG-*b*-PLA micelles was slower than that from drug solution, and sustained. Pharmacokinetic studies showed decreased total clearance and volume of distribution of alpinumisoflavone, whereas area under the curve (AUC) and bioavailability were significantly increased by incorporation in mPEG-*b*-PLA micelles. In vivo toxicity assay revealed that alpinumisoflavone-loaded mPEG-*b*-PLA micelles had no severe toxicity. In conclusion, we prepared an intravenous (i.v.) injectable alpinumisoflavone formulation, which was solubilized using mPEG-*b*-PLA micelles, and determined their physicochemical properties, pharmacokinetics, and toxicity profiles.

## 1. Introduction

*Cudrania tricuspidata*, also known as the silkworm thorn and storehousebush, is a thorny and perennial tree of the family Moraceae, which is widespread in East Asia. It has multiple nutritional and medicinal applications, so this tree has been used as a source of traditional medicine for the cure of some conditions such as contusions, insomnia, eczema, mumps, tuberculosis, and acute arthritis [1]. The fruit of *C. tricuspidata* is a berry that is rich in various active ingredients, including major functional ingredients such as polyphenols and flavonoids that possess diverse biological activities [2]. Despite its numerous nutritional and medicinal applications, the use of this fruit has been limited to simple oral intake including as juice, wine, jam, and folk medicine.

Alpinumisoflavone (AIF) is a pyranoisoflavone isoflavone, and one of the major bioactive ingredient derived from the unripe fruits of *C. tricuspidata* [3,4,5,6]. AIF exhibits numerous beneficial pharmacological activities such as osteoprotective [7], antibacterial [8], estrogenic [9], anti-metastatic [10], atheroprotective [11], and antioxidant [12] effects. Moreover, considerable evidence has shown that AIF possesses in vitro and in vivo anticancer activities [3,13]. Tongshun et al. showed that AIF suppressed metastasis and tumor growth of clear-cell renal cell carcinoma (CCRCC) by increasing miR-101 expression by inhibiting the Akt signaling pathway [10]. Namkoong et al. revealed that AIF suppressed the nuclear factor (NF)-κB pathway and extracellular signal-regulated kinases (ERKs)/mitogen-activated protein kinase (MAPK) and induced apoptosis of lung cancer cells [3]. AIF is also known to attenuate lipopolysaccharide-induced acute lung injury by regulating the effects of antioxidation and anti-inflammation both in vitro and in vivo [12]. Gao et al. [4] found that AIF impaired the metastatic potential of human melanoma (A375 and SK-MEL-1) cells by upregulating cell differentiation, as assessed based on melanin content, protoporphyrin IX accumulation, and tissue transglutaminase activity [4]. Interestingly, AIF regulates the expression of estrogen-sensitive genes associated with cholesterol clearance (cytochrome p450 7a1 [*Cyp7a1*], scavenger receptor class B member 1 [*Scarb1*], and low-density lipoprotein receptor [*Ldlr*]) and cholesterol synthesis (apolipoprotein A1 [*Apoa1*] and estrogen receptor 1 [*Esr1*]), and promotes the formation of bile acid and high-density lipoprotein (HDL) cholesterol, indicating its potential estrogenic activities [9]. However, despite the pharmacological usefulness of AIF, pre-clinical and clinical application of AIF has been restricted because of some limitations such as poor water solubility and a lack of pharmaceutical formulation studies. The intravenous (i.v.) formulation of poorly soluble drugs requires the use of solubilizing agents such as ethanol (EtOH), Tween 80, Cremophor EL^®^, and other solvents; however, these excipients are toxic to normal healthy cells [14,15]. For example, Taxol^®^, a solubilized paclitaxel dosage formulation for intravenous (i.v.) injection, contains EtOH and Cremophor EL^®^, which causes severe toxicities such as neutropenia, peripheral neurotoxicity, and life-threatening hypersensitivity reactions [16]. Moreover, Cremophor EL^®^ induces fatal hypersensitivity reactions in approximately 3% of patients with breast cancer despite pre-treatment with antihistamine and corticosteroid [17]. 

Several advanced methods for the solubilization of poorly soluble drugs were recently developed as alternatives to toxic solubilizing agents, including nanomicelles [18], solid dispersions [19], nanosuspensions [20], microemulsions [21], and liposomes [22]. Among them, polymeric micelles, which are round-shaped nano-vehicles consisting of amphiphilic block copolymers (ABCs), have been investigated as effective drug nanocarriers [23]. When exposed to water, ABCs self-assemble in a process driven by the interaction of hydrophobic blocks [24]. The hydrophobic inner part can contain water-insoluble materials, whereas the hydrophilic outer part interfaces with water outside. This feature of ABC micelles provides many benefits to poorly soluble drugs for pharmaceutical application. First, as mentioned above, ABC micelles display an amphiphilic property, which is appropriate for encapsulating poorly soluble drugs in the micelle core, enabling solubilization as mentioned above [25]. Second, ABC micelles are biocompatible and biodegradable with little or no toxicity, and are stable in aqueous media [26]. It is also believed that renal excretion and non-specific capture by the reticuloendothelial system can be avoided [27]. Third, ABC micelles are very small sizes (<100 nm) and display improved vascular permeability at the target site. Lastly, preparation of micelles is relatively simple, thus it is rather practical and has potential to scale-up. 

In this study, we aimed to design an i.v. formulation using unripe *C. tricuspidata* fruits. To do this, we extracted active component AIF from unripe *C. tricuspidata* fruits and developed a preparation method for an i.v.-injectable liquid formulation. Furthermore, we optimized the AIF-injectable formulation by preparing several polymeric micelles for the solubilization of AIF, and compared the physicochemical properties: particle size, poly-dispersity index, and encapsulation efficiency. We then selected the best formulation and determined its release, pharmacokinetic, and toxicity profiles with the aim of producing a novel and potent micellar formulation for the clinical application of AIF.

## 2. Materials and Methods

### 2.1. Materials and Reagents

Methoxy poly(ethylene glycol)-*b*-poly(d,l-lactide) (mPEG[4k]-*b*-PLA[2.2k]) was purchased from Advanced Polymer Materials Inc. (Montreal, QC, Canada). Soluplus^®^ was obtained from BASF (Ludwigshafen, Rhineland-Palatinate, Germany). EtOH and acetonitrile (ACN) were purchased from Fisher Scientific Ltd. (Waltham, MA, USA). Distilled water (DW) was purchased from Tedia (Fairfield, OH, USA). Methanol (MeOH) was purchased from Honeywell Burdick and Jackson (Ulsan, Korea). Genistein was purchased from LC Laboratories (Woburn, MA, USA). Pluronics^®^ F127, ethyl acetate (EtOAc), and Cremophor EL^®^ were purchased from Sigma-Aldrich Corp. (St. Louis, MO, USA). All other reagents were of analytical grade or better.

### 2.2. Methods

#### 2.2.1. Collection of Unripe *C*. *Tricuspidata* Fruit and Isolation of AIF

The unripe *C. tricuspidata* fruits were collected from the herb garden in Chungbuk National University, Cheongju, Republic of Korea and identified by professor Mi Kyeong Lee, who is specialized in natural products. The AIF was extracted from the fruits using a previously reported method [2]. Briefly, 2.8 kg of the unripe fruits was pulverized and extracted twice with 75% EtOH. The EtOH extract (508.2 g) was suspended in H_2_O and then partitioned successively with solvents of increasing polarity to obtain *n*-hexane, dichloromethane (CH_2_Cl_2_), EtOAc, and *n*-butanol (*n*-BuOH) fractions. The CH_2_Cl_2_ fraction (CF, 44.6 g) was subjected to Sephadex LH-20 and eluted with MeOH to obtain 11 subfractions (CF1–CF11). Subfraction CF7 was purified by recrystallization from *n*-hexane-CH_2_Cl_2_ (1:1) to afford AIF (6.6 g).

#### 2.2.2. High-Performance Liquid Chromatography (HPLC) Analysis

**Figure 1:**
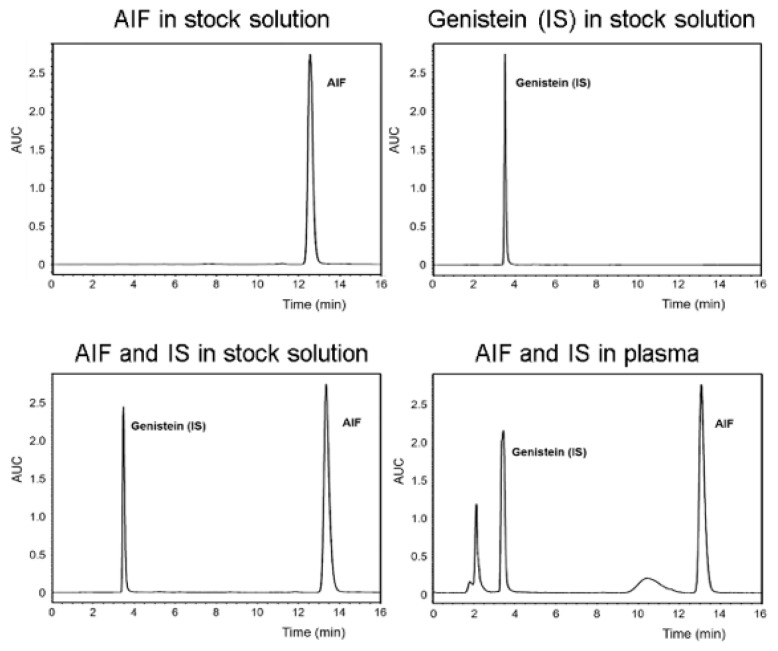
Representative chromatograms of alpinumisoflavone (AIF) and genistein (internal standard [IS]) in stock solution and biological sample.

The concentration of AIF in samples obtained from in vitro and in vivo assays in this study was determined using a Waters high-performance liquid chromatography (HPLC) system (Waters, Milford, MA, USA) equipped with Waters 2695 separations module and Waters 2996 photodiode array detector. The Fortis C18 chromatography column (5 μm, 4.6 × 250 mm) was used and maintained at 30 °C during the analysis. AIF and genistein (internal standard) were eluted using the isocratic mode with a mobile phase composed of ACN/water (70:30, *v*/*v*), which was freshly prepared for each run and degassed before use. The sample injection volume was 10 μL and flow rate was 1.0 mL/min for the mobile phase. The retention times of genistein and AIF were 3.4 and 12.7 min, respectively and their concentrations were calculated by comparing the peak areas with the standard curve (Figure 1).

#### 2.2.3. Determination of AIF Solubility

The solubility of AIF in DW or representative solubilizing agent was measured using a previously reported method [28]. To determine the solubility of AIF in water and a Cremophor EL^®^/EtOH mixture (representative solubilizing agent commercially used in Taxol^®^), AIF was added to 1 mL water, 100% EtOH, 50% Cremophor EL^®^/50% EtOH until saturation occurred. The solution was vortexed and centrifuged at 13,000 rpm for 5 min. The supernatant was filtered using a 0.2 µm regenerated cellulose filter (Corning Inc., Corning, NY, USA). The filtered clear solution was 100-fold diluted and 10 μL was injected into the HPLC system to determine the solubility.

#### 2.2.4. Preparation of AIF-Loaded Micelles

**Figure 2:**
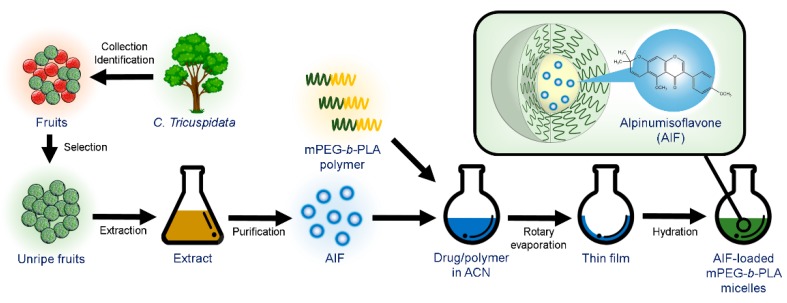
Extraction of alpinumisoflavone (AIF) from unripe *C. tricuspidata* fruits and preparation of AIF-loaded polymeric micelles using thin-film hydration method.

AIF-loaded polymeric micelles were prepared from various polymers using the thin-film hydration method [29]. Briefly, 6 mg AIF and various amounts of polymers (Pluronics^®^ F127 or mPEG(4k)-*b*-PLA[2.2k]) were dissolved in 1 mL ACN. For Soluplus^®^, 6 mg of AIF and various amounts of Soluplus^®^ were dissolved in a mixture of 0.8 mL MeOH/0.4 mL ACN. The drug–polymer mixture was added into round-bottomed flask and the solvent was evaporated using a rotary evaporator (EYELA, Bohemia, NY, USA) at 60 °C for 10 min under reduced pressure to obtain a thin film. After complete evaporation, the film was hydrated with 1 mL DW for 30 min to obtain a clear micelle solution. The micelle solution was then centrifuged at 13,000 rpm (Hanil Science lnc., Gimpo, Korea) for 5 min and filtered using a sterile regenerated cellulose filter (0.2 µm pore size) to remove the unincorporated drug and polymer (Figure 2).

#### 2.2.5. Physicochemical Characterization of Micelles

The particle size of the AIF-loaded micelles was measured using a dynamic light-scattering (DLS) device (Otsuka Electronics, Osaka, Japan). The prepared micelle solution was 10-fold diluted before determination. Drug-loading (DL, %) and encapsulation efficiency (EE, %) of the AIF-loaded micelles were determined using HPLC analysis and were calculated using the following equations:

DL% = Weight of drug in micelles/weight of feeding polymer and drug × 100

EE% = Weight of drug in micelles/weight of feeding drug × 100

The results of each sample analysis were expressed as the mean ± standard deviation (SD) of three separate experiments.

#### 2.2.6. In Vitro Drug Release Assay

The in vitro release behavior of AIF in the micelles was assessed using the dialysis method with phosphate-buffered saline (PBS, pH 7.4) as the release medium, over 336 h. In brief, samples of AIF-loaded mPEG-*b*-PLA micelles (5 mg/mL) and AIF solution (5 mg dissolved in Cremophor EL^®^:EtOH, 50:50, *v*/*v*) as the control were inserted into a pre-wetted dialysis membrane bag (MWCO 20 kD), which was tied and placed in 2.0 L release medium on a hot plate stirrer at 37 °C. The release medium was replaced with fresh medium after 8, 48, 120, 168, 216, and 288 h. At predetermined time intervals (0, 2, 4, 6, 8, 24, 48, 72, 168, 240, and 336 h), 20 μL of the sample was collected, 10-fold diluted with ACN, and the concentration of AIF was measured using HPLC analysis. Three experiments were conducted.

#### 2.2.7. Pharmacokinetic Study

All animal experiments were approved by the Institutional Animal Care and Use Committee (IACUC) of Chungbuk National University (CBNUR-1141-17, 24 October 2017). Male, Sprague-Dawley rats (7-week-old) were purchased from Orient Bio Inc. (Seongnam, Korea) and used for all animal experiments. The rats were housed in ventilated cages with free access to water and food. The rats in each group were i.v. injected with AIF (20 mg/kg) in 25% Cremophor EL^®^/EtOH (AIF solution) and AIF-loaded mPEG-*b*-PLA micelle. Despite its toxicity, Cremophor EL^®^/EtOH was used as a solubilizing agent for AIF solution. Blood samples were obtained from the retro-orbital plexus of the rats 5, 15, 30, 60, 120, and 240 min after administration and centrifuged at 3000 rpm for 5 min to obtain plasma samples, which were immediately frozen and stored at –70 °C until analysis. A non-compartmental model was used for the pharmacokinetic analysis to calculate relevant parameters: area under the concentration-time curve (AUC), total clearance (CL_t_), apparent volume of distribution (V_d_), and initial blood concentration (C_0_) of AIF after injection of each formulation.

#### 2.2.8. Biodistribution Study

The biodistribution study was conducted after i.v. injecting rats with the various formulations. At 8 h after administration, the rats were euthanized using carbon dioxide (CO_2_) and the liver, kidneys, spleen, heart, lungs, and muscle tissues were dissected. The tissue samples were washed in saline, wiped with paper towels to remove excess fluid, weighed, and then stored at −70 °C until analysis.

#### 2.2.9. Pre-Treatment of Biological Samples for HPLC Analysis

The frozen biological samples were thawed at room temperature. A 200 μL aliquot of the plasma sample was extracted with MeOH and 50 μL genistein (IS) was added, followed by centrifugation for 5 min at 13,000 rpm. The supernatant was filtered through a regenerated cellulose filter (0.2 µm pore size) and 10 μL of the sample was injected into the HPLC system for analysis [30]. The amount of AIF in the liver, kidney, spleen, heart, lung, and muscle samples was measured using homogenization method [31]. Briefly, the tissues were homogenized with a glass Potter-Elvehjem-type homogenizer with a Teflon pestle. After extraction of 200 μL of 20% homogenate with MeOH, the concentration of AIF in the supernatant was determined as described above.

#### 2.2.10. In Vivo Toxicity Assay

Five groups of mice (*n* = 3 per group) were used to evaluate in vivo toxicity. The rats in each group were i.v. injected with Dulbecco’s phosphate-buffered saline (DPBS, control), AIF (20 mg/kg) in 100% Cremophor EL^®^/EtOH solution, AIF (20 mg/kg) in 50% Cremophor EL^®^/EtOH solution, AIF (20 mg/kg) in 25% Cremophor EL^®^/EtOH solution, and AIF-loaded mPEG-*b*-PLA micelles (20 mg/kg). The body weight changes of all groups were monitored once every two days for a total of 14 days. The i.v. injections of AIF solution and micelles were carried out on day 0, 4, and 8. The toxicity of formulations was defined as loss of body weight loss (over 10%), abnormal behavior, signs of discomfort, or death. The body weight changes were normalized and displayed as percentages. In the toxicity test, rats judged to have moribund conditions were euthanized [32].

#### 2.2.11. Statistical Analysis

All data are expressed as the means ± SD. Distribution parameters are expressed as the means ± standard error (SE). Statistical analyses were performed using the Student’s *t*-test and statistical significance was accepted at *p* < 0.05 or < 0.01 (95% and 99% confidence levels, respectively).

## 3. Results and Discussion

### 3.1. Isolation and Identification of AIF

**Figure 3:**
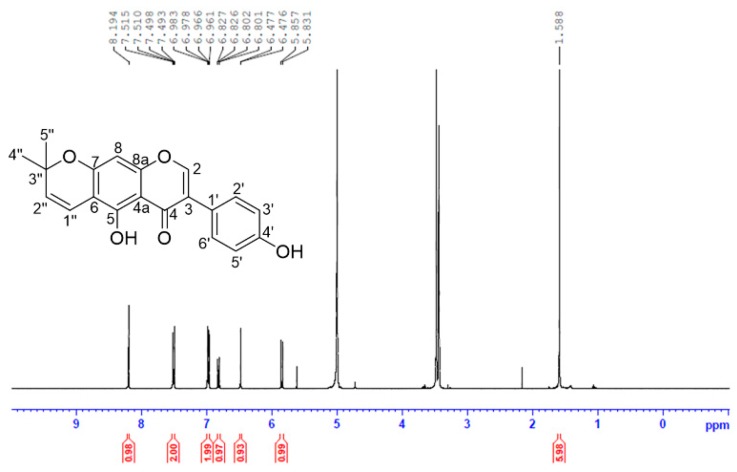
Isolation and nuclear magnetic resonance (NMR) imaging analysis of alpinumisoflavone (AIF) extracted from unripe fruits of *Cudrania tricuspidata.*

The isolated AIF from extract of unripe *C. tricuspidata* fruits was identified as follows: yellow crystal; UV (MeOH) λ_max_: 226, 282 nm; ESI-MS *m/z* 337 [M+H]^+^; ^1^H-NMR (MeOH-*d*_4_, 400 MHz) *δ*_H_ 8.19 (1H, s, H-2), 7.50 (2H, dd, *J* = 8.8, 2.0 Hz, H-2′, 6’), 6.97 (2H, dd, *J* = 8.8, 2.0 Hz, H-3′, 5′), 6.81 (1H, d, *J* = 10.0 Hz, H-1″), 6.47 (1H, s, H-8), 5.84 (1H, d, *J* = 10.0 Hz, H-2″), 1.59 (6H, s, H-4″, 5″) ppm (Figure 3).

### 3.2. Determination of AIF Solubility

Table 1 shows the solubility in water, Cremophor EL^®^/EtOH ratio (5:5, *v*/*v*), and Cremophor EL/EtOH ratio (0:10, *v*/*v*). The measured solubility of AIF in water was only 0.178 ± 0.121 μg/mL, confirming its poor water solubility. The solubility of AIF when dissolved in representative solubilizing agent (Cremophor EL^®^ and EtOH in half volume ratio) was 82.143 ± 9.989 mg/mL, indicating that the solubility could be elevated by using solubilizing agents, but the toxicity of these solubilizing excipients becomes a problem when designing an intravenously injectable formulation [16,33,34,35].

### 3.3. Physicochemical Characterization of AIF-Loaded Micelles

**Figure 4:**
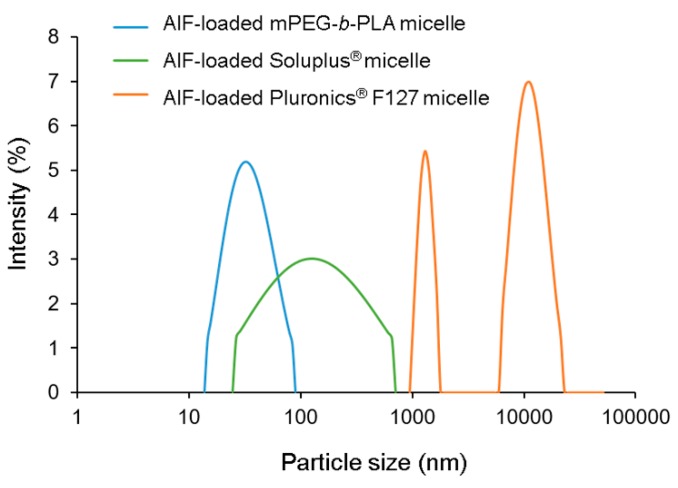
Representative size distribution profiles of alpinumisoflavone (AIF)-loaded mPEG-*b*-PLA micelles, AIF-loaded Soluplus^®^ micelles, and AIF-loaded Pluronics^®^ F127 micelles.

The EE (%), DL (%), and particle diameters of the AIF-loaded micelles prepared using various polymers (mPEG-*b*-PLA, Pluronics^®^ F127, and Soluplus^®^) are shown in Table 2. As demonstrated in the solubility section, measured solubility of AIF in water was very low (0.178 ± 0.121 μg/mL), indicating that a negligible portion (less than 0.01%) of the drug could exist in the aqueous phase of micellar solution. Furthermore, the unincorporated drug and/or polymer precipitates were removed by the centrifugation and filtration during the process. In this point of view, encapsulation of AIF in the polymeric micelles was well-characterized, as previous researches have done so far [16,17,36]. AIF-loaded mPEG-*b*-PLA and AIF-loaded Pluronics^®^ F127 micelles showed relatively high EE (39.5–81.0%) and DL (2.24–4.59%) values, whereas values of AIF-loaded Soluplus^®^ micelles were very low (AIF concentration, <0.1 mg/mL). In the DLS analysis, the average particle diameter of AIF-loaded mPEG-*b*-PLA micelles was 28.0–34.1 nm while those of other micelles prepared using Pluronics^®^ F127 or Soluplus^®^ were not detectable or showed multiple distribution with high (>0.5) polydispersity index (PDI) because of precipitation or abnormal conditions (Figure 4). The <100 nm particle size of the micelles indicates that phagocytosis and uptake by the reticuloendothelial system (RES) could be avoided, thereby prolonging its systemic circulation [37]. Considering the high EE and DL values and appropriate particle diameter, the mPEG-*b*-PLA polymer was selected for solubilization of AIF. At 135 and 150 mg, mPEG-*b*-PLA showed no significant differences in the EE, DL, and particle size. To minimize a potential unexpected effect of the excipient, the lower amount (135 mg) of the mPEG-*b*-PLA polymer and 6 mg AIF were the fixed amounts used in the preparation of AIF micelles, which will be used in further in vitro and in vivo studies.

### 3.4. In Vitro Drug Release Profile

**Figure 5:**
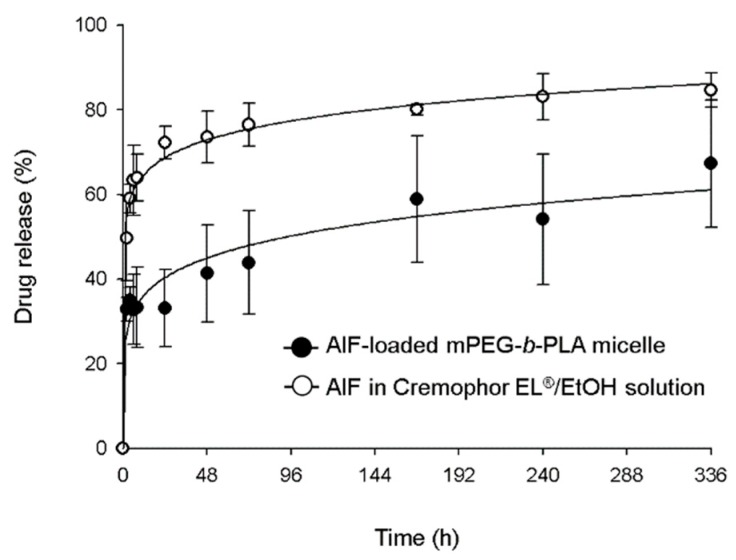
In vitro release profile of alpinumisoflavone (AIF) from mPEG-*b*-PLA micelles and Cremophor EL^®^/EtOH solution at 37 °C.

In vitro drug release profiles of AIF-loaded mPEG-*b*-PLA micelle and AIF dissolved in Cremophor EL^®^/EtOH solution are shown in Figure 5. The drug release of AIF-loaded mPEG-*b*-PLA micelles and AIF solution reached 32% and 63%, over 6 h, respectively. At 336 h, 84.6% of AIF was released from AIF solution, while 67.3% of AIF was released from AIF-loaded mPEG-*b*-PLA micelle, indicating that 

AIF in the mPEG-*b*-PLA micelles was released slower than that in the solution. This result indicated that the micelle carrier not only solubilizes poorly soluble drugs, but also restricts the rapid release of drugs and this is presumably due to intermolecular interaction of the hydrophobic drug and the hydrophobic segment of the polymer. These results showed that the micelle carrier not only solubilized the poorly soluble AIF, but it was also slowly released [38].

### 3.5. Pharmacokinetics of AIF in Rats

**Figure 6:**
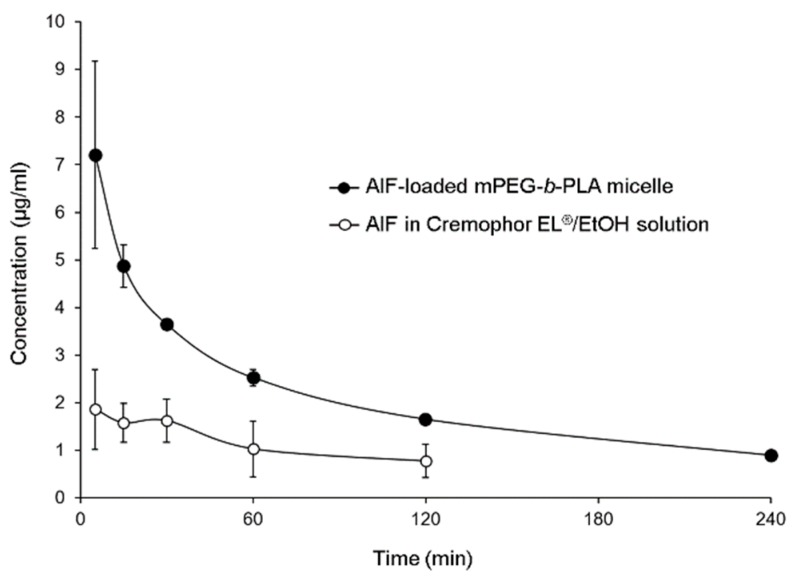
Alpinumisoflavone (AIF) plasma concentration time profile after intravenous (i.v.) administration of AIF-loaded mPEG-*b*-PLA micelles and AIF dissolved in 25% Cremophor EL^®^/ethanol (EtOH) solution.

The mean plasma concentration time profiles of AIF in mPEG-*b*-PLA micelles or 25% Cremophor EL^®^/EtOH solution are shown in Figure 6. The plasma concentration of AIF decreased rapidly after intravenous injection of AIF-loaded mPEG-*b*-PLA micelles or AIF solution in rats. However, it was found that the plasma concentration of AIF at 4 h after injection of AIF solution was lower than limitation of detection (LOD), while concentration of AIF at 4 h after injection of AIF-loaded mPEG-*b*-PLA was still detected in the plasma. The long circulation time of the micelles and sustained release of AIF contributed to the long retention of AIF in the blood. Pharmacokinetic parameters were calculated using a non-compartmental model. As shown in Table 3, when AIF was encapsulated in mPEG-*b*-PLA micelles, the CL_t_ and V_d_ values of AIF were decreased. The AUC and bioavailability (BA, %) values were significantly higher with AIF-loaded mPEG-*b*-PLA micelles than with the AIF solution (* p < 0.05). These results indicated that mPEG-*b*-PLA micelles significantly increased blood circulation time and decreased plasma clearance of AIF.

### 3.6. Biodistribution of Micelles in Rats

**Figure 7:**
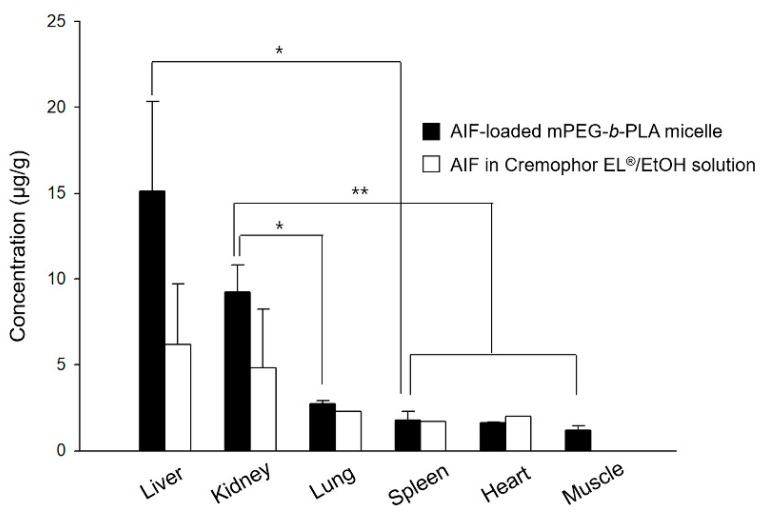
Mean concentration time profiles of alpinumisoflavone (AIF) in each tissue within 8 h after intravenous (i.v.) administration of AIF-loaded mPEG-*b*-PLA micelles and AIF dissolved in 25% Cremophor EL^®^/ethanol (EtOH) solution to rats (* *p* > 0.05, ** *p* < 0.01).

The drug amounts of AIF in major organs at 8 h after i.v. injection of AIF-loaded mPEG-*b*-PLA micelles or AIF dissolved in 25% Cremophor EL^®^/EtOH solution were determined and displayed in Figure 7. The amount of AIF (μg/g) in each tissue after i.v. injection of drug solution decreased in the following order of magnitude: the liver > kidney > lung > heart > spleen > muscle, while that after injecting the AIF-loaded mPEG-*b*-PLA micelles was: the liver > kidney > lung > spleen > heart > muscle. This finding confirmed that the AIF amount in both the solutions and mPEG-*b*-PLA micelles was high in the liver, which is a RES-related organ. However, a considerable amount of AIF also accumulated in non-RES organs, such as the kidneys, indicating that the tissue specificity of the micelles might also influence absorption by the RES system, as suggested by a previous study [39]. Overall, the mPEG-*b*-PLA micelles play a role as a vehicle of AIF, and the drug amount in major organs such as the liver, kidney, and lung were assumed to be relatively higher than that obtained with the drug solution, presumably because of the long circulation time in the blood [40,41].

### 3.7. In Vivo Toxicity Assay

**Figure 8:**
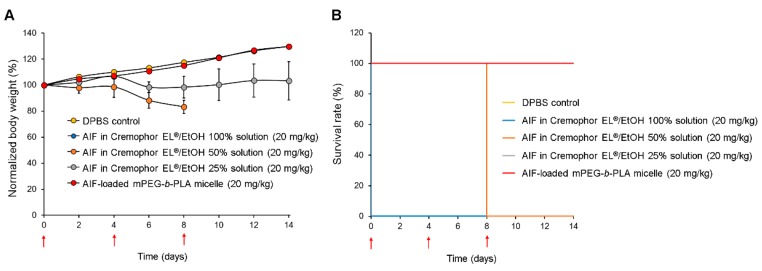
Relative body weight change and survival rate of rats after multiple intravenous (i.v.) injections of DPBS control, alpinumisoflavone (AIF) dissolved in 100% Cremophor EL^®^/ethanol (EtOH) solution, AIF dissolved in 50% Cremophor EL^®^/EtOH solution, AIF dissolved in 25% Cremophor EL^®^/EtOH solution, and AIF-loaded mPEG-*b*-PLA micelles on day 0, 4, and 8. (**A**) Relative daily body weight change. (**B**) Survival rate demonstrated using Kaplan–Meier plot [42]. Red arrows indicate intravenous injections of the formulations.

Figure 8 shows body weight changes of the rat groups after multiple injections of DPBS (control), AIF dissolved in 100% Cremophor EL^®^/EtOH solution, 50% Cremophor EL^®^/EtOH solution, 25% Cremophor EL^®^/EtOH solution, and mPEG-*b*-PLA micelles. As demonstrated in Figure 8A, rats in the groups treated with AIF dissolved in 50% Cremophor EL^®^/EtOH or 25% Cremophor EL^®^/EtOH solution exhibited significantly lower mean body weight increase than that in the control group. This shows that even after dilution to minimize the solubilizing agent levels, the Cremophor EL^®^/EtOH used as representative solubilizing agent was still toxic. In contrast, the mean body weight of rats in the AIF-loaded mPEG-*b*-PLA micelle group increased at a similar level to that of the control group, showing the absence of toxicity. All rats in the group treated with AIF dissolved in 100% Cremophor EL^®^/EtOH solution died immediately after i.v. administration, indicating the fatal toxicity of the solubilizing excipients (Figure 8B). In the group treated with AIF dissolved in 50% Cremophor EL^®^/EtOH solution, all the rats died 8 days after administration, presumably due to the cumulative toxicity of the solubilizing agents. In contrast, rats in the groups treated with DPBS (control), AIF-loaded mPEG-*b*-PLA micelles, and AIF in 25% Cremophor EL^®^/EtOH solution showed a 100% survival rate for two weeks. All the toxicity data indicate that multiple i.v. injections of AIF exerted no severe toxicity to rats even when mPEG-*b*-PLA micelles were used for solubilization.

## 4. Conclusions

In conclusion, we designed an i.v. formulation using *C. tricuspidata*. For this, we extracted AIF, a major compound of unripe *C. tricuspidata* fruits, and developed a preparation and solubilization method for a liquid dosage formulation using mPEG-*b*-PLA polymeric micelles. In addition, the physicochemical, pharmacokinetic, and toxicity properties of the AIF-loaded mPEG-*b*-PLA micelles were evaluated in vitro and in vivo. The nano-sized particles and slow release profile of the formulation prolonged the blood circulation of the drug after systemic administration, leading to increased bioavailability of AIF. Therefore, our designed micelle formulation warrants further pre-clinical investigation in preparation for subsequent evaluations in clinical trials for various pharmaceutical applications of AIF. We also expect the formulation to contribute to the added value of *C. tricuspidata* for medical use.

## Figures and Tables

**Table 1:** Solubility of alpinumisoflavone (AIF) dissolved in water and Cremophor EL^®^ /EtOH.

Solvent	AIF Solubility (μg/mL)
DW	0.178 ± 0.121
Cremophor EL^®^/EtOH (5:5, *v*/*v*)	82,143 ± 9989
Cremophor EL^®^/EtOH (0:10, *v*/*v*)	32,500 ± 6802

**Table 2:** Characteristics of alpinumisoflavone (AIF)-loaded micelles (*n* = 3, mean ± standard deviation [SD]).

Formulation	Polymer Amount Used (mg)	AIF Amount Used (mg)	AIF Final Concentration (mg/mL)	Encapsulation Efficiency (EE %)	Drug Loading (DL %)	Particle Size (nm)	Polydispersity Index (PDI)
AIF-loaded Pluronics^®^ F127 micelle	100	6	4.63 ± 0.08	77.2 ± 1.4	4.37 ± 0.08	1385 ± 430	0.45 ± 0.21
	135	6	4.86 ± 0.50	81.0 ± 8.4	4.59 ± 0.47	9993 ± 8616	0.60 ± 0.36
	150	6	4.52 ± 0.02	75.4 ± 0.3	4.27 ± 0.02	1102 ± 1170	0.59 ± 0.45
AIF-loaded Soluplus^®^ micelle	100	6	0.01 ± 0.01	0.24 ± 0.23	0.01 ± 0.01	138 ± 143	0.39 ± 0.18
	135	6	0.01 ± 0.00	0.09 ± 0.05	0.01 ± 0.00	N.D. ^a^	N.D. ^a^
	150	6	0.06 ± 0.09	0.94 ± 1.45	0.05 ± 0.08	78.7 ± 6.0	0.02 ± 026
AIF-loaded mPEG-*b*-PLA micelle	100	6	2.37 ± 2.16	39.5 ± 36.0	2.24 ± 2.04	28.0 ± 1.8	0.15 ± 0.05
	135	6	4.39 ± 0.09	73.1 ± 1.4	4.14 ± 0.08	34.1 ± 1.8	0.21 ± 0.02
	150	6	4.50 ± 0.15	74.9 ± 2.5	4.24 ± 0.14	31.2 ± 5.5	0.21 ± 0.01

^a^ N.D., not detectable.

**Table 3:** Pharmacokinetic parameters of alpinumisoflavone (AIF) after intravenous (i.v.) injection of AIF-loaded mPEG-*b*-PLA micelles and AIF dissolved in 25% Cremophor EL^®^/ethanol (EtOH) solution.

Parameters	AIF in 25% Cremophor EL^®^/EtOH Solution	AIF-Loaded mPEG-*b*-PLA Micelle
AUC ^a^ (min·μg·mL^−1^)	326 ± 168	876 ± 103 *
C_0_^b^ (μg·mL^−1^)	1.89 ± 0.63	7.86 ± 1.79
CL_t_^c^ (mL·kg^−1^·min)	94.6 ± 56.1	23.1 ± 2.7
V_d_^d^ (mL·kg^−1^)	13903 ± 5274	2940 ± 867
BA ^e^ (%)	100 ± 51	269 ± 18 *

^a^ AUC, area under the curve; ^b^ C_0_, plasma concentration at time 0; ^c^ CL_t,_ total clearance; ^d^ V_d_, volume of distribution; BA^e^, bioavailability, * *p* < 0.05.
